# The Urethral Hanging Theory: A Unified Biomechanical Model for the Pathophysiology and Treatment of Female Urinary Incontinence

**DOI:** 10.1007/s00192-025-06391-9

**Published:** 2025-11-29

**Authors:** Bo S. Bergström

**Affiliations:** 1 Years from 1988 to 2000, chief of the Department of Obstetrics & Gynecology, Mora Hospital, Lasarettsvägen 35, 792 51 Mora, Sweden; 2 Years from 2000 to 2006, overlege at the department of Obstetrics & Gynecology, Nordfjord Hospital, N-6771 Nordfjordeid, Norway; 3 Stockholm, Sweden

**Keywords:** Female urinary incontinence, Differential mobility, Differential descent, Funneling, Pathophysiology, Physical laws

## Abstract

**Introduction and Hypothesis:**

The pathophysiology of female urinary incontinence (UI) is often considered complex and poorly understood, partly due to the limitations of prevailing theories such as the integral theory and the hammock theory, both of which contradict established physical laws. This article introduces the urethral hanging theory (UHT) as a unified biomechanical model that explains stress urinary incontinence (SUI), mixed urinary incontinence (MUI), intrinsic sphincter deficiency (ISD), and idiopathic urge urinary incontinence (UUI) as a continuum of funneling-related pathologies.

**Methods:**

Anatomical analysis, physical principles, published work by others, clinical experience, and mental simulations were used to develop the model. The UHT attributes symptoms to differential mobility—a mechanical imbalance—between the urethra and the bladder neck, resulting in varying degrees of funneling during physical strain.

**Results:**

The theory provides a physically consistent framework that explains symptom variation across different types of UI and unifies them with a single biomechanical continuum. This framework also offers guidance for individualized surgical interventions.

**Conclusion:**

If validated clinically, the UHT could substantially improve diagnostic precision and treatment outcomes for female urinary incontinence.

## Discussion

**Fig. 1:**
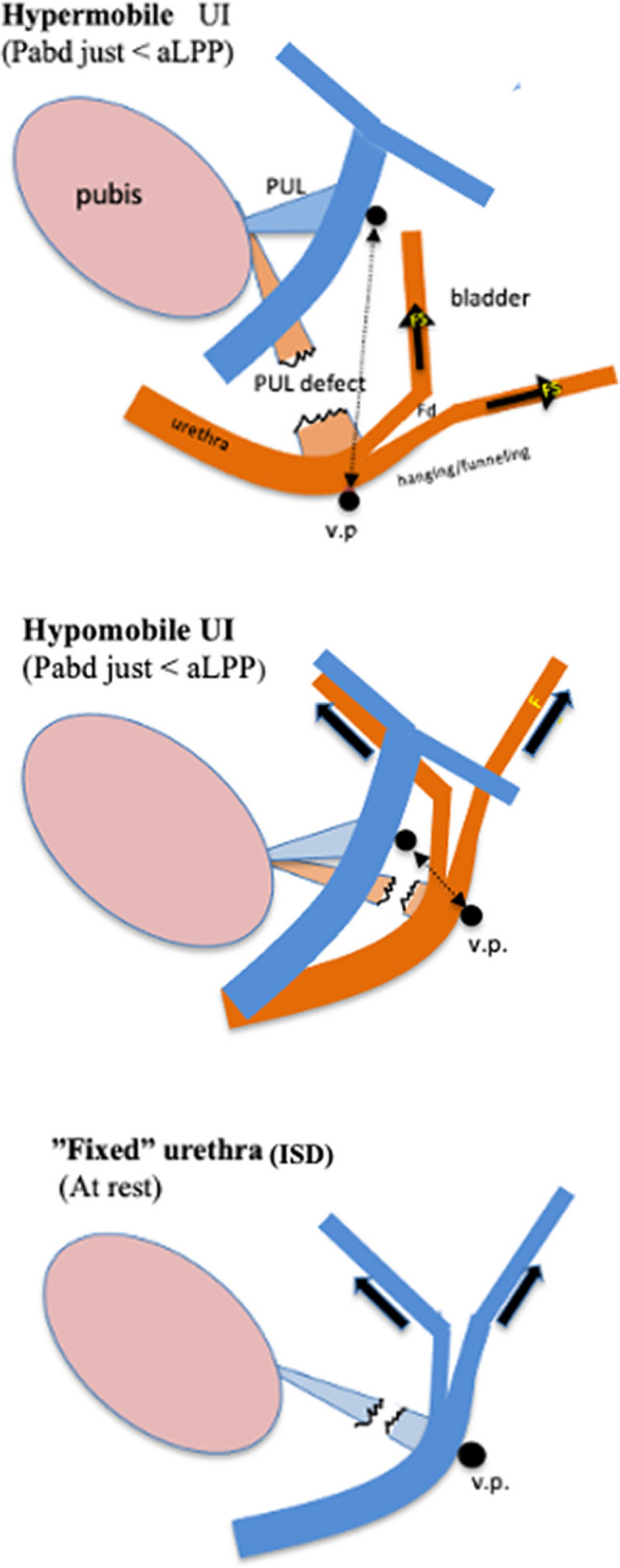
Demonstration of urethral hanging/forced funneling in hypermobile, hypomobile, and “fixed” types of SUI. The suburethral vaginal wall is pressed downward by intra-abdominal pressure and the proximal urethra is pulled open (forced funneled) by hanging on a less mobile BN. In the illustrated cases, no urine leakage occurs because the short funneling does not extend into the urethral high-pressure zone (HPZ). However, the forced distension of the richly innervated BN and proximal urethra may provoke urgency symptoms. It also shows the importance of the “therapeutic window” in choosing between a tension-free suburethral support and a lifting support. In cases with a minimally mobile bladder neck (“fixed” urethra) – where forced funneling occurs even at rest – a suburethral tension-free tape provides marginal, if any, benefit. In these cases, the proximal urethra at the vp must be lifted above its resting position; it should be obvious that hanging/funneling existing even at rest cannot be corrected by an elastic tension-free tape loosely placed under the posterior urethral wall. Lifting is also required in cases with a less hypomobile urethra that is not hanging at rest. This is because the use of tension-free vaginal tape (TVT) or transobturator tape (TOT) is associated with low cure rates, as the downward distance for the urethra to reach a hanging position is short, and a high Pabd makes the TVT and TOT sway downward slightly due to their elasticity. A TOT, in particular, sways downward because it is similar to a 5–8 cm long horizontal hammock that is laterally fixed on soft tissues. This is in contrast to a TVT, which forms a tight vertical loop that adheres to the lower part of the bony pubic body postoperatively. To create a lift without risk of obstruction, the “TVT technique” can be employed to insert one tuned tape into the paraurethral tissue on each side of the vp, or alternatively to elevate the proximal urethra by broadly folding the pubocervical fascia at the vp and supporting the plicated fascia with a tension-free suburethral tape (TVT); the plicated fascia creates a broad cushion between the urethra and the tape that prevents obstruction. Abbreviations: PUL, right posterior pubo-urethral ligament which attaches to the PCF. The “fractured” PUL represents schematically a defective PUL not specifically a split PUL; blue, urethra at rest; brown, urethra during stress; black arrow, therapeutic window (tw); Fs, pulling force that shears apart the urethral walls; Fd, outflow distending force; aLPP, abdominal leak point pressure; vp, vaginal point 15 mm from the BN. The distance between the vp at rest and the vp at aLPP defines the “therapeutic window.” A TVT located within this window during stress is curative. Clinically, the tw can be estimated by holding a fingertip a short distance under the vp at rest and asking the woman to perform a slow Valsalva maneuver. The maximum “curative” distance corresponds to the tw In hypermobile, hypomobile, and “fixed” types of SUI, the tw is large, small, and nearly zero, respectively. In women with urge urinary incontinence (UUI), there is no immediate leakage during stress, no measurable aLPP and no detectable t.w.

Many authors claim that the pathophysiology of female stress urinary incontinence (SUI) is not fully understood, often citing the complex interplay of factors involved in urethral closure. However, this perspective overlooks simpler biomechanical explanations rooted in anatomy and physics (Fig. 1). The two most widely cited theories of SUI—the Integral Theory (IT) by Petros and Ulmsten (1990) and the Hammock Theory (HT) by DeLancey (1994)—contradict fundamental physical laws. The IT rejects Pascal’s law in favor of a musculo-elastic mechanism, while the HT claims that, during stress, the transmission of abdominal pressure (Pabd) to the proximal urethra is delayed, which directly violates Pascal’s law. Moreover, the HT posits that the bladder neck is pushed open when bladder pressure exceeds urethral pressure. This contradicts the law of elastic collision, as a closed internal meatus cannot be pushed open—it must be pulled open.

Mid-urethral sling (MUS) surgery, based on the IT, has shown little improvement in failure or complication rates over three decades. One-year objective and subjective failure rates remain around 20% and 40%, respectively [1]. Nevertheless, the same “one-size-fits-all" procedure is still routinely performed. The TVT procedure has nearly a 100% cure rate in women with hypermobile SUI but shows poor results in those with hypomobile or fixed types of SUI. Classical TVT surgery does not differentiate between subtypes of SUI, nor does it tailor treatment accordingly.

Hypermobile SUI comprises 80% to 90% of cases and typically responds well to surgery, almost regardless of tape positioning. However, the remaining 10% to 20% of cases, with hypomobile or fixed types of SUI, account for most objective failures. Additionally, many women who are objectively cured still experience de novo or persistent urgency and frequency symptoms. These symptoms, along with eventual voiding issues, contribute to the 40% subjective failure rate. An accurate theory should be able to distinguish between appropriate and inappropriate surgery—which both the IT and HT fail to do.

**Fig. 2:**
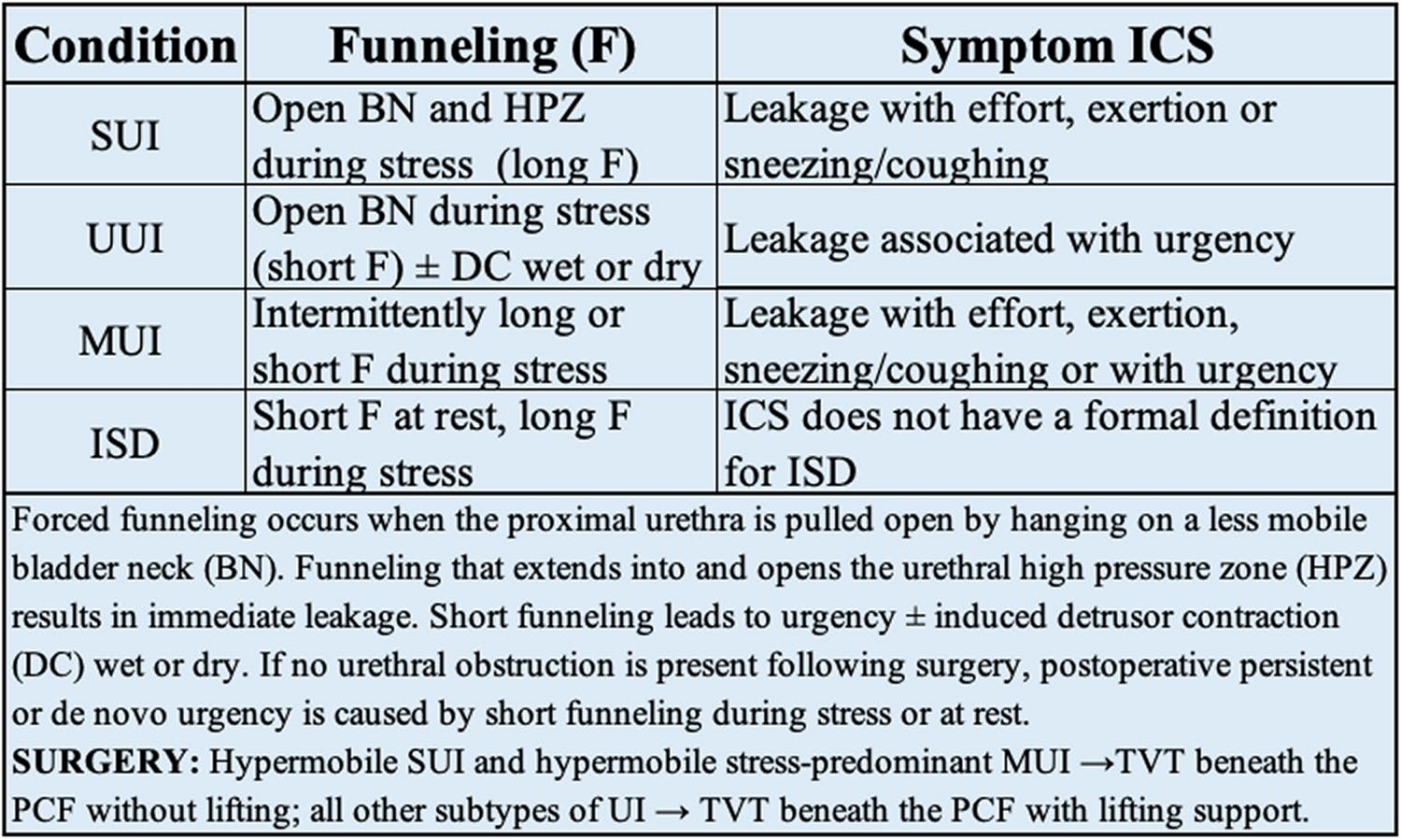
Diagrammatic representation of bladder neck and urethral funneling patterns in various forms of urinary incontinence, highlighting their pathophysiological significance and relevance to surgical management

Urinary incontinence (UI), which affects millions of women globally, is typically categorized into SUI, MUI, ISD, and idiopathic UUI. Although traditionally treated as distinct conditions, clinical overlap and unclear pathophysiology challenge this segmentation. This article introduces a unifying framework for female incontinence based on the urethral hanging theory (UHT) by Bergström (2016) [2–4]. UHT proposes that differential mobility and descent between the urethra and bladder neck (mechanical imbalance) during physical stress lead to varying degrees (lengths) of bladder neck and proximal urethral funneling. This triggers a spectrum of symptoms ranging from stress-induced leakage to involuntary detrusor contractions [5, 6]. The concept that UI subtypes share a common origin is supported by earlier work. Petros and Ulmsten, in their 1990 article, proposed that both stress and urge symptoms stem from the same anatomical defect, and several subsequent researchers have similarly suggested that UI subtypes may represent a single disease spectrum [7–10]. Emerging evidence indicates that urgency and UUI may result from abnormal afferent signaling involving the urethra and its supporting structures, rather than from a primary detrusor pathology. Imaging and urodynamic studies by Rinne et al. [11] and Pirpiris et al. [12] support the association between SUI and excessive mid-urethral mobility, most pronounced 15–16 mm from the bladder neck [11, 12]—the position defined by the UHT. In healthy, continent women, mid-urethral mobility is less than bladder neck mobility. UHT integrates SUI, MUI, ISD, and idiopathic UUI into a continuum of funneling-related pathologies determined by support integrity. All UI subtypes result from forced funneling caused by differential mobility rather than absolute urethral mobility, where the richly innervated BN and proximal urethra are pulled open (Fs) and distended (Fd) by hanging on a less mobile BN (Fig. 2). When funneling extends into and opens the urethral high-pressure zone (HPZ)—i.e., long funneling, immediate leakage occurs, as in SUI. Short funneling, confined to the bladder neck and proximal urethra, leads to urgency, with or without induced detrusor contractions (wet or dry), as in UUI. Funneling, which varies in length between short and long, manifests as MUI. Short funneling present even at rest combined with long funneling during stress, as in ISD, is associated with severe urgency in addition to leakage under minor stress, as in the fixed type of SUI which according to UHT corresponds to ISD (“stove pipe urethra”). Without forced funneling, urine leakage does not occur, irrespective of how low the urethral pressure may be. Accordingly, the maximum urethral closure pressure at rest (MUCP) is not the critical factor in UI [4].

### Treatment according to UHT

Since all four subtypes of UI arise from a common underlying dysfunction—mechanical imbalance caused by relative motion between two structures under equal pressure—they should be managed accordingly [13–15]. Surgical treatment aims to prevent asymmetric mobility and descent of the proximal urethra relative to the BN. Sling placement should therefore be tailored to the specific mobility pattern of the urethra–bladder neck complex rather than applied uniformly across all women. The therapeutic window (t.w.; see Fig. 1) determines whether suburethral support alone is sufficient or whether additional lifting is required. In hypermobile SUI and hypermobile stress-predominant MUI—cases with a large t.w.–the tape (width 11 mm) should be placed tension-free below the PCF, starting 1 cm from the bladder neck, meaning the tape’s center is positioned at the vaginal point (vp), 15.5 mm from the bladder neck. Here, “below” refers to the space between the PCF and the vaginal mucosa.

In hypomobile or fixed types of SUI (ISD), – cases with a small or nearly zero t.w. where the urethra is already or nearly hanging at rest – the proximal urethra at the vp should be lifted above its resting position. To create this lift without risking obstruction, the proximal urethra should be elevated by broadly folding the pubocervical fascia (PCF) at the vp and supporting the plicated fascia with a tension-free suburethral tape (TVT). If the PCF is thick and well-developed, slight lifting support can be provided by a tensioned TVT without folding the fascia. Alternatively, the TVT technique can be used to elevate the urethra by inserting a tuned tape into the paraurethral tissue on each side of the vp. On the basis of clinical experience, vaginal folding is rarely required when the TVT is placed below the PCF and tensioned just enough to prevent leakage during a cough stress test. In women with UUI, there is no immediate stress leakage, no measurable abdominal leak point pressure (aLPP), and no detectable t.w. Because the exact onset of urethral funneling during urethral descent cannot be clinically assessed, the same lifting support described above—PCF folding/TVT–should be applied. The same principle also applies to women with urge-predominant MUI. All four subtypes of UI may be cured by providing lifting support. However, in hypermobile SUI and hypermobile stress predominant MUI, lifting is not necessary.

Positioning the tape below, rather than above, the PCF increases the risk of tape expulsion through the vaginal wall but reduces the risk of a more serious complication—erosion into the urethra.

The rationale for lifting support is to “stop urethral hanging,” not to increase abdominal pressure transmission, which is always 100% [14, 15]. Classical TVT surgery instructs that the tape should be placed above the PCF and always without tension, as a tensioned tape compresses and obstructs the adjacent soft urethra. This explains the high failure rates of surgery in women with hypomobile and fixed types of SUI, where the urethra at rest is hanging or nearly hanging on a less mobile bladder neck—that is, those with a zero or small therapeutic window (Fig. 2). Given this, such women cannot be cured with an elastic, tension-free tape loosely placed under the posterior urethral wall. To achieve a cure, the margin to urethral hanging must be increased through a lifting support procedure. The IT misrepresents the pathophysiology of SUI and misguides surgical approaches for hypomobile and fixed types of SUI

When the tape is placed above the PCF, lifting support cannot be performed without causing urethral obstruction, as is repeatedly stated by Petros. Accordingly, UHT recommends slight lifting with the tape placed below the PCF, with or without fascial folding, as the key to treating hypomobile or fixed type SUI.

In the absence of urethral obstruction caused by surgery, persistent or de novo urgency is typically due to forced funneling, either at rest or during stress. This occurs when the tape is placed too loosely, too distally, or without lifting support [5].

Interestingly, a study by Petros shows that additional lifting support is necessary for some women with SUI. Up to 20% to 30% of patients required vaginal folding—tightening the hammock (“pinch”)—in addition to mid-urethral anchoring to prevent urine loss during coughing [3, 16, 17]. However, Petros did not identify these women as having the hypomobile or fixed type of SUI, nor did he describe vaginal folding as a lifting-support procedure. Instead, he proposed that the folding increased the contractile activity of the horseshoe-shaped rhabdosphincter. In October 2022, Petros introduced the urethral ligament plication (ULP) procedure, which employs two polyester sutures as a simpler treatment for SUI [18]. These sutures function by shortening the pubourethral ligaments, thereby elevating the urethra above its resting position. This approach thus abandons the classical tension-free tape technique in favor of a lifting-support principle. The ULP procedure has the potential to prevent hanging and forced funneling without increasing the risk of urethral obstruction. According to UHT, the ULP procedure may be effective for all subtypes of UI, although its long-term durability remains uncertain. Unlike a TVT supporting a folded PCF fascia, ULP sutures may cut through the tissue, and the neocollagen may stretch over time.

## Conclusions

UHT provides a biomechanical explanation for SUI. The model is simple, and the virtual modeling process allows for mental simulations to predict outcomes, such as different grades of urethral funneling under physical stress. Funneling is caused by differential mobility, rather than by differential pressure transmission or loss of ligament–muscle tension, as proposed by the HT and IT, respectively [14–16]. The urethra does not “leak because it’s weak”; it leaks because it’s hanging. The purpose of the tape is to prevent mechanical descent mismatch, and in cases requiring lifting, also to compensate for mesh elasticity. Since its proposal in 2016, the UHT has remained explicitly falsifiable; however, no imaging, urodynamic, or computational data have refuted it. Further validation now requires demonstration that urethral mobility exceeds bladder-neck mobility in women with funneling. UHT is both parsimonious and physically consistent. If confirmed clinically, it has the potential to redefine diagnostic and treatment approaches in female urinary incontinence while also providing a comprehensive explanation for Howard Kelly’s 1913 description of a gaping bladder neck—an early observation of the hanging/forced funneling phenomenon [19]. 

## Declarations

### Disclaimer

This article presents a novel explanatory framework whose clinical applicability has not yet been validated. The interpretations and surgical implications discussed are based on anatomical analysis, physical principles, clinical experience, and mental simulations, and should therefore be regarded as theoretical until confirmed through rigorous clinical research.
